# Hospital and Institutional News

**Published:** 1914-11-28

**Authors:** 


### HOSPITAL AND INSTITUTIONAL NEWS.

### SCOUTS HELP THE BOOT FUND.

On Thursday, November 19, a display was given at Bath by the St. Matthew's Church, Widcombe, Scouts in aid of The Hospital Belgian Boot Fund. It was organised by the Rev. R. W. B. Rees, who has sent us a cheque for £3 6s. 3d. and to whom we are greatly indebted for his example and action. The programme, which was entirely carried out by the Scouts, opened with the English and Belgian National Anthems, and consisted of various items of physical drill, gymnastics, stretcher drill, etc., interspersed with songs and recitations. It concluded with an exhibition of bridge-building, in which a bridge 4 feet high and of sufficient strength to carry the weight of twelve Scouts was built by the lads. During the evening the amount which had been raised by the effort was announced, as given above.

### PATIENT'S ATTACK ON A MEDICAL SUPERINTENDENT.

The condolences of all mental hospital workers will go out to Dr. Hetherington, the medical superintendent of the Londonderry Mental Hospital, in the terrifying ordeal through which he has just passed. According to Mr. Barrie, M.P., who brought the matter before the Londonderry Mental Hospital Committee, a violent patient in some way obtained possession of a knife and made a sudden attack with it upon Dr. Hetherington, who has been connected with the hospital for over forty years. He was stabbed in several places, but grappled with the patient, and, though his strength was failing rapidly, owing to loss of blood, he managed to hold his assailant safely until help was obtained and the lunatic was removed to safe custody. The incident is a notable illustration of the dangers which may always lurk in the path of a medical officer in a mental hospital. Although administration should be capable of preventing "accidents" like the above, as we all know they are extremely rare.

### SOLDIERS' STAY IN HOSPITAL: OFFICIAL INSTRUCTIONS.

In the case of some of the voluntary hospitals, with regard to Belgian soldiers, it is interesting to note that the War Office has issued further instructions with reference to the stay of wounded soldiers in hospital. It appears that the War Office desires that Belgian soldiers should be kept in hospital till fit for active service, and not be sent to convalescent homes. We understand that when they are fit for active service the embarkation officer at Folkestone, as well as the Belgian Military Bureau at 48 Sandgate Road, Folkestone, is to be communicated with, and arrangements will then be made for them to report there. On the other hand, men permanently unfit have to report to the Belgian Military Bureau, 10 Finsbury Square, E.C., when arrangements will be made for discharge from the Army. How far the military authorities are aware that this new plan may "hold up" hospital beds which have previously been set free by the assistance of convalescent homes is not apparent. In view, however, of the prospective demand for further beds as the war goes on, this official sanction of a lengthened stay may cause some difficulty and require readjustment.

### "CANVASSING WILL BE CONSIDERED A DISQUALIFICATION."

We wish committees of management would act up to their advertisements, and where the above clause is inserted have the public spirit to see that it is carried out. It may be urged that the committees who do not carry it out are self-condemned, but that is a poor consolation to a first-rate man who is supplanted by one who has canvassed, and, as is sometimes done, actually attributes his successful candidature to having disregarded the advertised prohibition. The consequence is that men of sufficiently high standard of honour to read these advertisements strictly according to their literal meaning are placed in a false position, and do not know what course they are expected to pursue. Is there any way in which the practice of disregarding the prohibition against canvassing can be effectually brought home to committees who fail to act on their pretensions and to men who have been cynically encouraged to laugh at those who strictly carry it out? This is a matter which lies well within the scope of the two associations of hospital officers, and if they would combine to place their views before the managers of hospitals generally, there can be no doubt that a most undesirable state of things could be put a stop to. In our judgment the British Hospitals Association and the Incorporated Association of Hospital Officers should insist on two points. The first is that a clause forbidding canvassing should be inserted in every advertisement of a vacant administrative appointment; and the second that every instance of a breach of the rule should be reported to the officials of the two bodies. We have very little doubt that if the first point were unanimously agreed to, every hospital committee would co-operate with the two associations to carry out the second. A unanimous expression of opinion on this head would make easy the public exposure of any breach, which is now more than any individual officer can be expected to undertake, especially since, as things are, the very men who disregard the prohibition when it is made are often those who claim to have benefited by it. We should be glad to have our readers' experiences in this connection.

### A DOUBLE LOSS TO THE SEAMEN'S HOSPITAL.

Death has deprived the *Dreadnought* Seamen's Hospital of two stalwart supporters in the persons of Captain Sir G. R. Vyvyan, K.C.M.G., and Captain Francis M. Ommanney, R.N. As befits those who work for the *Dreadnought*, both of these were distinguished sailors—the former was Deputy Master of the Trinity House, an office not perhaps fully understood by the general public. It is known that the Trinity House is responsible for the lighthouses and buoys round our coasts, but it is not known that the Deputy Master is really the head of the Elder Brethren of the Trinity House, the Master being always a Royal personage. Sir George Vyvyan joined the board of management of the *Dreadnought* twenty-six years ago. After rendering much assistance to the charity, he was promoted to the Vice-Presidents' list; but this did not end his close association with the work of the hospital, for he continued until his death an earnest worker on its behalf, and the old marine charity stands for all time the better for his connection with it.

### THE OMMANNEY FAMILY AND THE SEAMEN'S HOSPITAL.

CAPTAIN FRANCIS METCALFE OMMANNEY, R.N., whose death is referred to in the preceding paragraph, first joined the committee of management of the *Dreadnought* Seamen's Hospital in 1892, and brought with him a revival of the old association of the Ommanney family, several of his predecessors, including the well-known Arctic explorer, Admiral Sir Erasmus Ommanney, having held a seat upon the committee. As soon as Captain Francis M. Ommanney joined, he became one of the most active members, constantly visiting the various establishments and being practically never absent from the board meeting. At the end of fifteen years he became deputy chairman, and in this capacity worked in closest association with the chairman, Mr. Percival A. Nairne, whose labours in connection with the *Dreadnought* Hospital are so well known. A typical sailor, the patients in the care of the *Dreadnought* thoroughly understood him. His sympathies lay with the sick and the suffering rather than with medical education, which perhaps it may be said he tolerated. He did good work for the Seamen's Hospital Society, and his death is a great blow to it.

### BELGIAN MEDICAL STUDENTS IN LONDON.

THE London School of Tropical Medicine has offered the hospitality of the hostel attached to the school to medical graduates and students of Belgium who may be refugees in this country. Already one Belgian medical student, Dr. Brittlis, is in residence.

### MEDICAL TREATMENT OF ATTENDANTS.

THE difficulties created by the Insurance Act in regard to the medical attendance of institutional staffs are apparently still causing trouble in some of the Scotch mental hospitals. At a meeting of the Forfarshire County Insurance Committee vigorous criticism was made in respect of the medical treatment of the attendants at West Green Mental Hospital. Mr. J. R. Pratt, Birkhill, declared that much friction had been caused at West Green, which had only been settled by the judicious handling of the clerk, Mr. Hanick. The medical men, continued the speaker, seemed to think that they could draw their full fees for medical attendance and medicine, but immediately a man became seriously ill they turned him out, and he was left to be attended by anybody. Only in a mental hospital would such treatment be tolerated. The superintendent had shown him an agreement by which apparently they had a right to act in this way. The mental hospital staff were receiving the sixpence which, he understood, was only to be paid for domiciliary treatment, and they were not giving that treatment. Some clause, he concluded, should be inserted in the agreement to prevent attendants from being turned into the street. After further discussion, in which Mr. Pratt's assertion that the hospital's regulations said that the attendants must take a certain doctor or walk out was challenged by the chairman, Provost Moffatt, who said that the agreement did not compel the employees to take the doctor in the institution, the matter was referred with powers to the medical benefit sub-committee. Since in most institutions the terms of engagement include medical attendance, in England the complaint has been against the staffs being included in the insurance scheme at all. It was stated by the clerk at the above meeting that at Montrose Mental Hospital the fees are returned to insured persons because medical attendance is provided. But this does not meet the point that the provision of medical attendance becomes a grievance to the man who is limited compulsorily to his hospital's staff. It will, therefore, be interesting to see the conclusions at which the medical benefit sub-committee arrive.

### INFIRMARY MEDICAL OFFICERS AND ACTIVE SERVICE.

DR. NOCKOLDS, assistant medical superintendent at Lewisham Infirmary, has left to take charge of an Anglo-Belgian Red Cross hospital. As he is receiving a special salary for his services the board has decided to grant him leave of absence without salary. Dr. St. Johnstone, a former medical officer at the infirmary, has been appointed temporary assistant medical superintendent in his place, at a salary of £5 5s. a week with the usual residential allowances. He has been permitted with Mrs. St. Johnstone to take up his residence in the assistant medical superintendent's quarters.

### THE SALARIES OF INFIRMARY ASSISTANT MEDICAL OFFICERS.

OWING to the difficulty in obtaining assistant medical officers—repeated advertisements at the former inadequate salaries having failed to produce any candidates—the Paddington Board of Guardians recently decided to appoint two temporary medical officers at their infirmary at a salary of £250 per annum, with residential allowances, for a period of six months. In a letter sanctioning the proposal, the Local Government Board stated that they did so in view of the special circumstances, but that it must not be inferred that they will be prepared to sanction a similar rate of remuneration in the case of permanent officers. This pronouncement of the central authority is to be deplored. It is in striking contrast with its action in the matter of the new scale of salaries for medical officers recently adopted by the Metropolitan Asylums Board. That Board proposed a salary of £250 for their junior assistant medical officers, £300 for the second medical officers, and £350, rising to £400, for the seniors. This scale was sanctioned without any restrictions as to time. The Poor-Law Medical Service will never become efficient until the Local Government Board realises that the salaries must be revised until they are brought into line with those offered by other public authorities. Poor-Law medical officers have always been the worst paid and hardest worked of all the public medical services. Now that the supply of candidates, which has been growing smaller for years past, has finally ceased owing to the demands of the war, Boards of Guardians have been forced to adopt a more generous attitude. It should be the duty of the Local Government Board to encourage and not to deter them in their attempts to make the service more attractive.

### WHAT TO OMIT IN YOUR APPLICATIONS FOR PROMOTION.

The art of omission is the true art of application-making, for a letter of application is like a legal contract in this, that every additional word extends its scope. Moreover, in the case of a letter of application you are very obviously giving hostages to fortune, for your vanity will incline you to say the most of your achievements, while any indications of vanity will probably prove fatal to your chances. The only rule is the simple, but difficult, one to stick closely to facts that are indisputable. Do not say "I have a great gift for raising money": state the sums you have raised. Do not say "I showed His Majesty round the new wards," when every other member of the staff and board was there too. Senior men will smile at these suggestions; but it is not the senior men who apply for higher posts. Among juniors it is often believed that a turn of phrase, or a playing on the supposed idiosyncrasies of the committee to whom your application is being sent, may decide the day. But the catch-penny method must be fatal to applicants for the higher posts, where besides technical training that quality of large-mindedness which we call a sense of responsibility is required, and that only comes from a habit of mind to which catch-penny methods do not appeal. That is why terseness and brevity are the best recommendations in applications for promotion, but their importance is one which the senior men always have great difficulty in making their promising subordinates realise. Omit also imaginary qualifications, such as, for instance, membership of a club which purports to be a technical association, but is, in fact, only a popular edition of the accredited institute or society; such "second strings" seem to exist in all professions. When all these things have been omitted, or struck out on wiser thoughts, from your application form, you will be surprised how simple and forcible are the grounds of qualification which remain to you.

### WINSLEY SANATORIUM ADMINISTRATION SCHEME.

The committee of the Winsley Sanatorium, at the recent November meeting, were engaged in discussing the draft scheme for the future administration of the institution under the Charity Commissioners. A meeting of the board of management is about to be held to decide the final draft previous to its submission to the Commissioners. The affairs of the sanatorium have frequently been discussed in The Hospital, and it is to be noted that Dr. Bathe, the resident medical officer, who has stayed by request till a successor could be found, is now leaving, and the house committee have placed on record their thanks for "his kindness and attention to the patients at all times," and for his accession to their request to postpone his resignation.

### THE MANAGEMENT OF SANATORIUM PATIENTS.

Complaints having been recently made as to the treatment provided at the Daneswood Sanatorium, Woburn Sands, Bedfordshire, a sub-committee of the London Insurance Committee has investigated the complaints and finds that they are not substantiated. The sub-committee say that patients who were questioned stated definitely that they were perfectly satisfied with the attention and the treatment which they received from the staff, and on no occasion had there been any complaints as to the food supplied. Every care appeared to be taken to ensure that the food was of good quality and adequate in quantity, and the serious complaints made as to the unsatisfactory character of the food generally did not appear to be justified. The sub-committee further point out that in view of the type of patient admitted to the institution the staff are called upon to exercise considerable tact and discretion, and they are satisfied that the institution is well conducted and that the matron is suitable for the post and performs satisfactorily the difficult duties devolving upon her. The attention of the management committee has been drawn to certain matters of detail and discipline in which the sub-committee think that improvement might be effected, and they have every reason to believe that their suggestions will receive careful consideration. In this connection, by way of comparison, we may refer our readers to a letter on "Discipline in Convalescent Homes" which appears in our Correspondence columns this week.

### THE DIRECTOR OF THE MEDICAL SERVICE OF THE WELSH ARMY CORPS.

Mr. J. Lynn Thomas, C.B., of Cardiff, who has identified himself with all Welsh national movements, has been appointed Director of the Medical Service of the Welsh Army Corps. Up to his recent retirement he commanded an extensive practice as a surgeon throughout the Principality, whilst he has had military experience as a major in the Glamorgan Royal Garrison Artillery (1902

to 1912) and in the 2nd Welsh Field Ambulance, R.A.M.C. He was born in Cardiganshire on September 10, 1861, and educated first privately and afterwards at the London Hospital and Vienna. He obtained the F.R.C.S. in 1892. During his career he has held the positions of house surgeon to the London Hospital and assistant surgeon to the Dinorwic Quarries Hospital. His present appointments include: Honorary surgeon to King Edward VII.'s Hospital, Cardiff; consulting surgeon of the Royal *Hamadryad* Hospital, Cardiff; the Cardiff Dispensary, the Porth Hospital, the Bridgend Hospital, and King Edward VII.'s National Memorial Association. He rendered conspicuous services to his country as senior surgeon with the Welsh Voluntary Hospital in the South African War, for which he received a war medal with three clasps. He is a Justice of the Peace for the counties of Cardigan and Glamorgan, and also acted as High Sheriff for his native county in 1907. It will be remembered that a few months ago he placed his private hospital at Bedford House at the disposal of King Edward VII.'s Hospital, Cardiff.

### THE WELSH WAR HOSPITAL, NETLEY.

Work at the Welsh War Hospital, Netley, is now in full swing, and there is a constant turnover of patients. The majority of the men have been wounded in the limbs by shell, and though many have been seriously hurt only one death is recorded. About a quarter of the building has been converted into an officers' hospital, which has necessitated some reconstruction. Recreation rooms also are being provided. There have been numerous institutional and other visitors to the hospital.

### SANATORIUM BENEFIT AND THE POOR RATE.

Vigorous protests are being made against a suggestion of the Metropolitan Asylums Board to provide out of the rates money for the building, equipment, or maintenance of sanatoria for patients entitled to treatment under the National Insurance Act. In the annual report of the Board for last year it is pointed out that a Government grant will be available towards the cost of the provision of sanatoria, and the Local Government Board, who will be responsible for the distribution of the grant, have announced that three-fifths of the cost up to £90 per bed will be defrayed from this grant. The cost of treating insured persons will be met out of the funds provided by the Insurance Committee. The cost of treating dependants of insured persons, when undertaken by the Insurance Committee, so far as it is in excess of any surplus available from the Insurance Act Funds, will be met as to one-half by the Treasury when the remainder is paid out of the county rates, and the cost in respect of uninsured persons will be met in equal parts from the same two sources. The report proceeds to state that the question of the manner in which that part of the cost which in any case has to fall upon the rates in London should be raised does not appear to be very material, and it would probably simplify the procedure if the Board accepted the responsibility, and raised the amount in the same manner as the cost of the rest of their work is raised. It is maintained that although the above may be an easy way of meeting the deficit, it is a very unfair one, as the amount required will have to be included in the precepts of the Boards of Guardians, who would thus get the credit, or otherwise for the increased expenditure, whereas sanatoria treatment has nothing to do with them. It is urged as only reasonable that the National Health Insurance authorities should reimburse the Metropolitan Asylums Board the whole cost out of their funds instead of placing the burden on the ratepayers of London, who never anticipated that they would be called upon to pay anything towards what from the outset was stated to be a movement entirely free and independent of the poor rate.

### DEATH OF A HOSPITAL ARCHITECT.

Death has removed a prominent Leicester architect in the person of Mr. Stockdale Harrison. He was responsible for the construction of many well-known buildings, one of the latest being the "Faire" Hospital in Leicester, a report on which appeared in the columns of The Hospital of August 1 on the occasion of the visit of Sir Henry Burdett to Leicester.

### THE INFIRMARY'S RELATION TO THE MENTAL HOSPITAL.

There are many reasons why the Poor-Law infirmary should continue to act as the receiving homes for pauper cases of lunacy, but, once certified, the case should be as quickly as possible transferred to the specialised institution provided. It is only when we have one authority working in close co-operation with the infirmary medical superintendents that the full scope of the problem will be realised and the necessary provision made. At present there is too much diffusion of effort and too little combination. Each authority is working along its own lines, with no thought, and often with no knowledge, of what is being done by the others or of the difficulties each is meeting with and trying to solve. As we pointed out last week the L.C.C. should be made the sole authority for providing institutional treatment for lunacy and mental deficiency in London.

### A BISHOP ON MEDICAL MISSIONS.

The Bishop of St. Albans, speaking at the recent annual meeting of the Melanesian Mission, held at the Church House, Westminster (the Bishop of Rochester, President of the English committee, in the chair), referred to the hospital which has been built at Maratovo, Solomon Islands, in memory of the late Rev. H. Welchman, where an English doctor and two qualified nurses are already in charge. He said there could not have been a finer memorial to Mr. Welchman than that hospital; there could not have been a finer continuance of that great healing work which he did for so long among those islanders. There they had an admirably equipped hospital, and they were likely very soon, by the aid of an improved vessel, to be the means of bringing patients there from other islands, and to be able to take the doctor himself from time to time to do medical work in other islands as well. When they looked back at the history of missions during the past century, it seemed to him that the growth of medical missions was one of the most prominent features. To-day there was no mission which had not its medical side. The importance of the medical side in mission work has always been insisted on by The Hospital, and our readers will recall that an illustrated article on the Welchman Hospital at Maratovo appeared in The Hospital of April 25 last.

### THE NEW STATIONERY OFFICE AS A HOSPITAL.

At the request of the War Office it is announced that the Government have placed at the disposal of the War Joint Committee of the British Red Cross Society and the St. John Ambulance Association the new Stationery Office in Stamford Street as a Red Cross hospital. The institution is expected to comprise 1,650 beds; and we understand that the equipment of the building and the provision of the staff will be in the hands of the Joint Committee, while the Office of Works is to adapt the structure to hospital purposes. Known as the Waterloo Hospital, sums of £25 are asked for to provide beds and its necessary hospital equipment, thus providing to the lay mind, if it would think the matter out, some idea, by comparison, of what items the cost per bed in the civilian voluntary hospitals has to cover. Military hospitals are proverbially larger than civil ones, but we rather doubt whether, in an improvised building, not designed for hospital purposes, and consequently administratively difficult, so large a number of beds as 1,650 can be dealt with adequately; since even in buildings specially planned for the treatment of the sick, it is found in practice that even half this number is about as difficult to superintend as need be. This criticism, of course, raises the question as to whether there is any natural limit to the number of beds which should constitute a modern efficient hospital. We believe that there is, and if we were asked for the highest number in round figures we should say 700, or even less. Hospitals, like hotels, beyond a certain size lose in administrative efficiency what they gain in impressiveness. Some considerations on this point are contained in our critical description of Glasgow Royal Infirmary on page 197.

### ILLUSTRATED APPEALS.

The notes and comments on raising hospital income in war-time, and our correspondent's request for information on what he described as "the five points of a successful appeal," have produced several communications, and among them an article on hospital finance and income-raising generally, which argues that appeals are only a minor, though an important, part of voluntary finance. Almost simultaneously we received a copy of the annual appeal of the Royal Hospital for Incurables, Putney Heath, which in its customary booklet form we have often had pleasure in praising for originality both of matter and presentation. This year, under the title of "Our 'At Home,'" an account is given of what happened at this annual function on Founder's Day last year, with plenty of illustrations by Mr. Bernard Hugh, the most imaginative, as also the most poster-like of which, is certainly the one entitled "The Home on the Hill," which would surely have been worthy of a place on the cover, even if the present wording had to be altered somewhat. It is no doubt extremely difficult to keep an equally high standard of interest and attractiveness in these rightly ambitious annual appeals; and, therefore, having praised some extremely good ones of late years, we may say frankly that the present is not the best which has been done. People are generally more interested in programmes of future events than of past ones, and consequently in taking last year's Founder's Day as a text the compilers of this appeal had an initial difficulty. Still, in his best illustration, already mentioned, Mr. Bernard Hugh has achieved a design which will survive the year for which it was drawn. Being pleasantly printed and arranged, the reputation of previous years will no doubt give success to the work which has gone into this year's appeal pamphlet.

### "GLAMORGAN AND MONMOUTH HOSPITAL FOR THE FRENCH ARMY."

A fund has been started in Cardiff for the equipment of a fifty-bed hospital to be called the "Glamorgan and Monmouth Hospital for the French Army." An hotel in France with the necessary accommodation has been placed at the disposal of the promoters, and of the £2,000 required, the greater part has already been subscribed. Miss Sybil Corbett and Madame Raoul Vaillant de Guelis are the joint honorary secretaries, and Monsieur Neltner, the French Consul at Cardiff, is the honorary treasurer. Dr. H. G. Cook, M.D., B.S., F.R.C.S., an assistant honorary surgeon at King Edward VII.'s Hospital, Cardiff, has consented to go out in charge of the hospital. A letter has been received from the ex-Empress Eugénie giving her full approval of the committee's undertaking, and wishing the hospital every success.

### THE POOR-LAW STANDARD AND INFIRMARY TRAINING SCHOOLS.

At a meeting of the Bath Guardians last week the administration of the Frome Road House Infirmary came up for discussion, owing to the difficulty of obtaining probationers. Mr. T. Wills, chairman of the house committee, pointed out that the committee recommended the appointment of a non-resident whole-time medical officer at a salary of £350, rising to £400. Were this done, the chairman declared that the institution would rank henceforward as a training school, by complying with the requisite conditions of possessing 100 beds and a full-time medical officer. After other speakers had objected that the proposal would mean getting rid of the medical men who had done duty for a long period, Dr. Curd was appealed to. Admitting this difficulty he nevertheless supported the appointment of a full-time medical officer on the lines laid down in the resolution. This was carried, and Dr. Duckett was appointed to the post, with which he is to combine the duties of medical officer to the six scattered homes, public vaccinator, and lecturer to the probationers. We confess that a full-time medical officer who is also non-resident cannot be regarded, from the modern hospital standard, as satisfactory. It is the perpetuation under a slightly modified form of the old system, for no medical officer who is non-resident can, or does, have that undivided administrative control over the sick wards of the institution which is essential to the welfare of the patients, and, we may add, to the ultimate reputation of the institution as a training school. So long, in fact, as complete separation is not secured infirmaries are only infirmaries in name, and the post of non-resident whole-time medical officer is one of the anomalies which characterise all schemes which fall short of this *desideratum*.

### AN INVALID BEQUEST TO A HOSPITAL.

The question whether the mortage debentures of certain Australian land companies can be validly bequeathed to voluntary hospitals and charities was raised in the Chancery Division of the High Court last week. One of the institutions concerned was the Sussex County Hospital at Brighton. Briefly, it appeared that the testator's residuary estate had been left on trust for his son for life, and after the son's death upon trust for his nephew, save for so much of the said trust premises as might by law be applicable for charitable legacies, which sum he left upon trust in equal shares for the Sussex County Hospital and one other institution. The nephew died in the testator's lifetime; the testator's son, his sole heir-at-law, in 1913. Part of the testator's residuary estate consisted of £6,000 in debentures in two Australian land companies, neither of which had any interest in land in England beyond the leasehold of their London offices. There was evidence that by the law of all the Australian States, charities could acquire land in Australia as freely as private individuals, and that any moneys forming part of the estate of the testator charged on land in Australia could be validly given by his will to charity. Mr. Justice Neville, in the course of his judgment, said that he could not distinguish this case from others where a legacy to a charity of money charged on pure and impure personalty had been held void under the Mortmain Act of 1888. Here the moneys secured by these debentures were charged on a mixed fund of real and personal estate, although it was quite true that the leasehold offices of the companies in London were of very little, if any, value. He was, therefore, of opinion that the charities were not entitled to these debentures.

### THE EMPLOYMENT OF BELGIAN PHARMACISTS.

A considerable number of Belgian pharmacists is reported among the refugees who have arrived in this country, and the proposal has been made that they should be employed temporarily in place of those assistants who have joined the new armies. While the Belgian diploma is not recognised in this country it is urged that there is no reason why this class of refugee should not be employed under the supervision of a British pharmacist, and indeed, in many instances, the language difficulty, quite apart from more cogent reasons, would compel some such supervision. But we feel it necessary to point out that, while sympathising deeply with the skilled professional refugees in this country, their employment, even temporarily, raises almost more difficulties than it would solve. Already in the case of organised labour the trade unions have felt compelled to urge that no refugee should be employed in any trade, so organised, except only while there is a shortage of British labour. Though the professions are not so well organised as the skilled trades, yet inevitably each professional association must take into consideration a similar safeguard for its members; and the difficulty resolves itself into finding a *modus vivendi* between merely supporting the professional refugee along with his fellows, and preventing them from disorganising the English branches of the profession to which they belong. We should be glad to publish the views of pharmacists on this question. Is it practicable to find them employment?

### THIS WEEK'S DRUG MARKET.

Attention is still centred on the opium market, and the upward tendency of prices of opium, morphine, and codeine continues. There has been rather more activity in quinine as a result of the removal of this drug from the list of articles of which the export is prohibited; a small advance in the price of quinine would not come as a surprise. Ergot of rye still has a downward tendency in price, and by waiting it might be possible to obtain better terms. Buyers are reluctant to pay the very high prices wanted for chamomiles; as shipments of Belgian chamomiles are expected from Holland it is not improbable that prices may presently be lower. Cocaine has a lower tendency in price, and although quotations are still substantially higher than they were immediately before the war, the present figure can hardly be considered excessive under the circumstances. Gentian root and digitalis leaves have a lower tendency.

### TO OUR READERS.

Contributions are specially invited from any of our readers to these columns. They should deal with topical subjects and news. They must be authenticated for the information of the Editor only. The minimum payment if published is 5s. There is no hard-and-fast rule as to space, but notes of about twenty lines in length are preferred.

